# Mechanotransduction in high aspect ratio nanostructured meta-biomaterials: The role of cell adhesion, contractility, and transcriptional factors

**DOI:** 10.1016/j.mtbio.2022.100448

**Published:** 2022-10-03

**Authors:** Khashayar Modaresifar, Mahya Ganjian, Pedro J. Díaz-Payno, Maria Klimopoulou, Marijke Koedam, Bram C.J. van der Eerden, Lidy E. Fratila-Apachitei, Amir A. Zadpoor

**Affiliations:** a Department of Biomechanical Engineering, Faculty of Mechanical, Maritime, and Materials Engineering, Delft University of Technology, Mekelweg 2, 2628CD, Delft, the Netherlands; b Department of Orthopedics and Sports Medicine, Erasmus MC University Medical Center, Doctor Molewaterplein 40, 3015GD, Rotterdam, the Netherlands; c Department of Internal Medicine, Erasmus MC University Medical Center, Doctor Molewaterplein 40, 3015GD, Rotterdam, the Netherlands

**Keywords:** High aspect ratio nanopillars, Osteogenic response, Mechanotransduction, Focal adhesion kinase, Rho-associated protein kinase, Yes-associated protein

## Abstract

Black Ti (bTi) surfaces comprising high aspect ratio nanopillars exhibit a rare combination of bactericidal and osteogenic properties, framing them as cell-instructive meta-biomaterials. Despite the existing data indicating that bTi surfaces induce osteogenic differentiation in cells, the mechanisms by which this response is regulated are not fully understood. Here, we hypothesized that high aspect ratio bTi nanopillars regulate cell adhesion, contractility, and nuclear translocation of transcriptional factors, thereby inducing an osteogenic response in the cells. Upon the observation of significant changes in the morphological characteristics, nuclear localization of Yes-associated protein (YAP), and Runt-related transcription factor 2 (Runx2) expression in the human bone marrow-derived mesenchymal stem cells (hMSCs), we inhibited focal adhesion kinase (FAK), Rho-associated protein kinase (ROCK), and YAP in separate experiments to elucidate their effects on the subsequent expression of Runx2. Our findings indicated that the increased expression of Runx2 in the cells residing on the bTi nanopillars compared to the flat Ti is highly dependent on the activity of FAK and ROCK. A mechanotransduction pathway is then postulated in which the FAK-dependent adhesion of cells to the extreme topography of the surface is in close relation with ROCK to increase the endogenous forces within the cells, eventually determining the cell shape and area. The nuclear translocation of YAP may also enhance in response to the changes in cell shape and area, resulting in the translation of mechanical stimuli to biochemical factors such as Runx2.

## Graphical abstract

**Figure:**
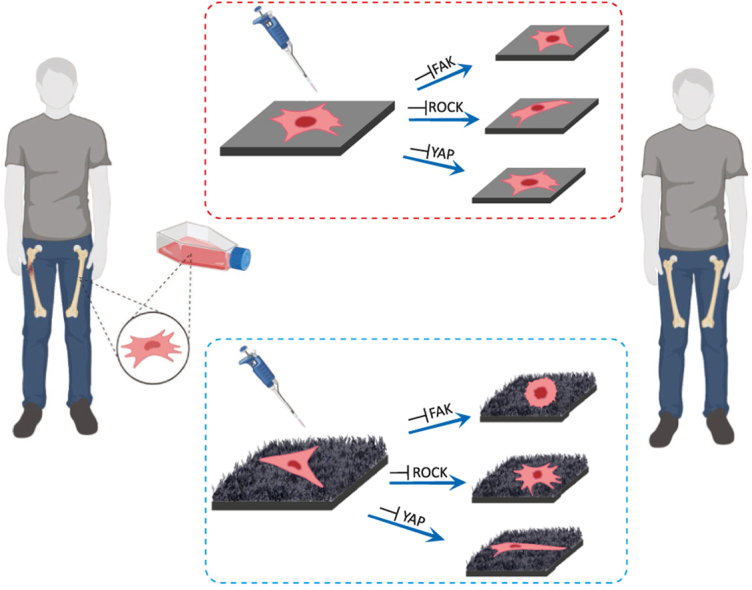

## 1 Introduction

Cell-instructive biomaterials, as a relatively new category of advanced functional materials, have expanded the horizon for tissue regeneration and cell therapy applications as they embody different functionalities that enhance their modulatory role with regard to the fate of the cells with which they interact [1,2]. In this regard, the effects of biophysical interactions between the stem cells and the biomaterial's surface on their phenotype and lineage commitment has been extensively studied in recent years [3,4]. Cells can sense the small-scale features of surfaces and transduce exogenous mechanical forces to their nuclei where these signals can be converted to different biochemical signals, eventually affecting the cell function [5]. Previous studies have revealed that the geometry (*i.e.*, shape, arrangement, and dimensions) of the surface features substantially contributes to the induction of a variety of responses in cells [6,7]. High aspect ratio nanostructured surfaces, in particular, offer remarkable potential for manipulating and or controlling not only the biomechanical environment of the cells but also their biochemical and bioelectric environments [8]. For instance, they can be exploited as 1) carriers for intracellular delivery or extraction by harmlessly penetrating the cell membrane [9,10], 2) a platform for investigating cell-material interactions including mechanotransduction pathways [4], and 3) highly bactericidal surfaces to combat biomaterial-associated infections [11]. The combination of the rare properties and functionalities displayed by these surfaces that result in unusual biological responses has framed them as meta-biomaterials [8,12].

High aspect ratio nanostructures come in different geometrical shapes (*e.g.*, pillars, cones, needles, *etc.*) and arrangements (*i.e.*, ordered or stochastic), and can be synthetically fabricated through a variety of subtractive or additive manufacturing techniques (*e.g.*, electron beam lithography (EBL), two-photon polymerization, dry etching, *etc.*) [8]. A recently reported fabrication process for generating high aspect ratio nanopillars on the Ti surface (as the most clinically-relevant material for orthopedic applications), also known as black Ti (bTi), is inductively coupled plasma reactive ion etching (ICP RIE) [13]. The effects of the processing parameters of ICP RIE on the geometry of the resultant nanopillars have been investigated before [14]. Rational changes in these parameters yield certain configurations of nanopillars that exhibit superior mechano-bactericidal properties [7,15]. The same surfaces also enhance matrix mineralization in murine preosteoblasts [7] and human adipose-derived mesenchymal stem cells (hASCs) [15]. Although different theories have been developed regarding the mechano-bactericidal actions of dry-etched surfaces, such as bTi [16], less is known about the exact mechanisms leading to osteogenic differentiation in cells. For instance, changes in the cell morphology and distribution of focal adhesions (FAs) have been observed on bTi surfaces, yet the role of subcellular components in the eventual commitment towards osteogenic differentiation has not been fully understood [7,13].

Integrin signaling is known to play an important role in sensing the mechanical forces generated as a result of the interactions of a cell with the extracellular matrix (ECM) [4,17]. To transduce the mechanical forces from the ECM to the nucleus, FAs play a mediatory role to connect membrane-bound integrins to the cytoskeleton. Focal adhesion kinase (FAK) is a major component of the FA complex that is mainly associated with the integrins’ β1 subunit and regulates the adhesion of cells to their underlying substrate. FAK is known to trigger and regulate multiple downstream pathways, which promote the expression of osteogenic markers [4,18]. Accordingly, silencing/inhibiting FAK has been shown to downregulate the expression of osteogenic markers [19,20]. Moreover, the dynamic remodeling of the cytoskeleton is associated with externally exerted forces as well as the endogenous forces generated within the cells [4,21]. Activation of RhoA from the Rho family of GTPases and its downstream Rho-associated protein kinase (ROCK) is essential for the phosphorylation of the myosin light chain (MLC) and promoting contractility via myosin II [21]. Therefore, the RhoA/ROCK pathway not only plays an important role in sustaining the integrity of the cytoskeleton and determining the cell shape but also affects the mechanical tensions sensed by the nucleus [21,22]. The inhibition of this pathway has also been shown to hinder the osteogenic differentiation of human adipose-derived mesenchymal stem cells (hASCs) [20]. In addition to cell adhesion and contractility, the role of transcriptional factors, such as Yes-associated protein (YAP) and co-activator with PDZ-binding motif (TAZ) in mechanotransduction has been a matter of interest [23,24]. For instance, YAP has been shown to control the FA assembly and cell shape upon translocation to the nucleus but its nuclear translocation is dependent on the RhoA/ROCK pathway [25].

**Fig. 1:**
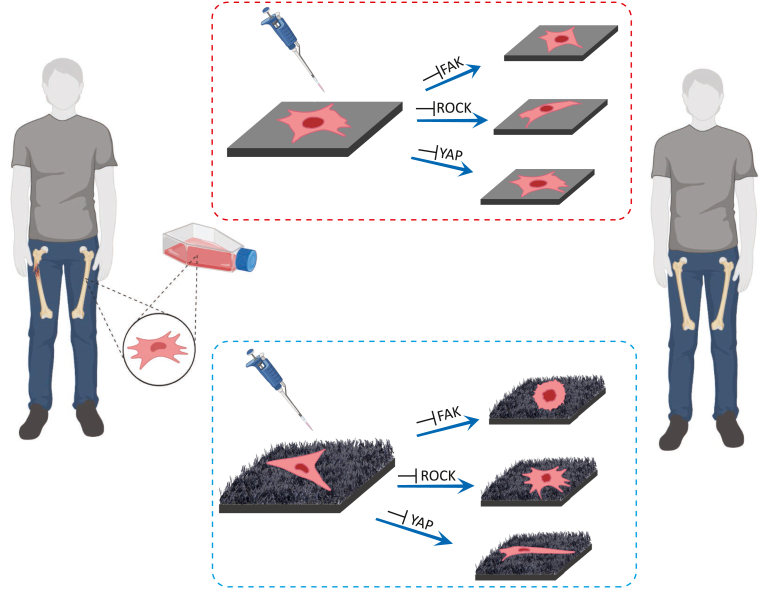
A schematic drawing of the experiments carried out in this study. Biomaterials developed for enhancing bone tissue regeneration may consist of conventional material (flat Ti) or meta-biomaterial (high aspect ratio bTi nanopillars) which exhibit different osteogenic potential. The cell responses to these surfaces and the intracellular mechanotransduction pathways that regulate them were investigated under normal or inhibitory conditions.

The potential intracellular mechanotransduction pathways triggered by high aspect ratio bTi nanopillars have not been studied before. Here, we investigate the possible intracellular pathways by which high aspect ratio bTi nanopillars promote the osteogenic differentiation of (stem) cells, with a focus on the role of cell adhesion, contractility, and transcriptional factors. We use a unique configuration of bTi nanopillars, previously shown to enhance the mineralization of murine preosteoblasts [7], to study the early adaptation of human mesenchymal stem cells (hMSCs) to such surfaces. We found that bTi surfaces enhance the expression of Runx2 in hMSCs compared to the flat Ti, which happens subsequent to changes in the cell morphology, the formation of FAs, and the nuclear localization of YAP in cells. Thereafter, we investigated three hypotheses, namely the osteogenic properties of bTi nanopillars are 1) FAK-dependent, 2) ROCK-dependent, and 3) YAP-dependent (Fig. 1). We then discuss the relationship between these hypotheses to highlight their potential interdependencies.

## 2 Materials and methods

### 2.1 Fabrication and characterization of high aspect ratio bTi nanopillars

A previously described ICP RIE protocol [7,14] was used to create high aspect ratio nanopillars on titanium surfaces. Briefly, annealed titanium foils with a thickness of 125 ​μm (99.96% purity, Goodfellow, UK) were cut to the size of a 4-inch (diameter ​= ​10.2 ​cm) silicon wafer and were polished with chemical-mechanical polishing (CMP Mecapol E460, France). After being cut into 8 ​× ​8 ​mm^2^ pieces, the surface of the titanium specimens (glued with a thermal paste atop a 4-inch quartz wafer as the carrier wafer) were etched with Cl_2_ and Ar gases using an ICP RIE machine (PlasmaLab System 100, Oxford Instruments, UK) under the following conditions: ICP source power ​= ​600 ​W, RF power ​= ​100 ​W, etching time ​= ​10 ​min, Cl_2_ flow rate ​= ​30 sccm, Ar flow rate ​= ​2.5 sccm, chamber temperature ​= ​40 ​°C, and chamber pressure ​= ​2.0 ​Pa. Once the etching process was concluded, the specimens were cleaned in acetone, ethanol, and isopropanol, respectively.

The etched surfaces were then imaged with a scanning electron microscope (SEM) (Helios NanoLab 650, FEI, US) from the top and 35° tilted positions. The height and the tip diameter of the nanopillars were then measured based on SEM images. Furthermore, the major chemical elements present on the surface of bTi specimens were identified by performing energy-dispersive X-ray spectroscopy (EDS) inside the SEM. Finally, the water contact angle of the specimens was measured by a drop shape analyzer (DSA 100, Krüss, Germany) as described before [7,14].

### 2.2 Investigating the response of hMSCs to high aspect ratio nanopillars

#### 2.2.1 Pre-culture of cells and cell seeding

hMSCs isolated from the bone marrow of healthy donors and expanded to the second passage were purchased from Lonza (#19TL329433, PT-2501, Lonza Bioscience, The Netherlands). The cells were expanded until the fifth passage in the alpha minimum essential medium (α-MEM) supplemented with 10% (v/v) fetal bovine serum, 50 ​μg/ml gentamicin, and 1.5 ​μg/ml amphotericin B (all from Thermo Fisher Scientific, US). 1 ​ng/ml fibroblast growth factor-2 (Bio-Rad, The Netherlands) and 0.1 ​mM ascorbic acid (Sigma-Aldrich, Germany) were freshly added to the refreshing expansion medium [26]. Prior to the cell culture, the flat Ti and bTi samples were sterilized by immersion in 70% ethanol and then exposure to UV light for 20 ​min. The samples were placed in a 48 well-plate (Greiner, Bio-One, The Netherlands) containing 300 ​μl of the culture medium in each well 2–3 ​min before seeding the cells. Upon reaching confluence, the cells were detached from the cell culture flask using 1X trypsin-EDTA solution (Thermo Fisher Scientific, US), were cultured on both flat Ti and bTi surfaces (1 ​× ​10^4^ ​cells per sample), and were incubated at 37 ​°C with 5% CO_2_ (Life Technologies, US). In the case of the experiments taking longer than 1 day, the α-MEM medium was supplemented with 0.1 ​mM ascorbic acid, 10 ​mM β-glycerophosphate, and 1 ​mM dexamethasone (all from Sigma-Aldrich, Germany) from day 2 onwards. All the experiments, described in the following sections included three samples per study group.

#### 2.2.2 Immunocytochemical analysis of hMSCs settled on high aspect ratio nanopillars

In order to evaluate the early-stage adaptation of hMSCs to bTi surfaces, the cells were stained for the actin filaments, nucleus, and focal adhesions after 1 day of culture. Briefly, after washing the samples with 10X PBS (Sigma-Aldrich, Germany), the cells were fixed using a 4% (v/v) formaldehyde solution (Sigma-Aldrich, Germany) and their membranes were permeabilized by adding 0.5% Triton X-100/PBS (Sigma-Aldrich, US) at 4 ​°C for 5 ​min. The samples were then incubated in 1% BSA/PBS (Sigma-Aldrich, Germany) at 37 ​°C for 5 ​min and were subsequently incubated in anti-vinculin mouse monoclonal primary antibody (1:100 in 1% BSA/PBS, Sigma-Aldrich, Germany) and rhodamine-conjugated phalloidin (1:1000 in 1% BSA/PBS, Thermo Fisher Scientific, US) for 1 ​h at 37 ​°C. The immunofluorescent staining process was followed by washing the samples thrice with 0.5% Tween-20/PBS (Sigma-Aldrich, US) and incubating them in Alexa Fluor 488, donkey anti-mouse polyclonal secondary antibody (1:200 in 1% BSA/PBS, Thermo Fisher Scientific, US) for 1 ​h at room temperature. The samples were washed again three times with 0.5% Tween-20/PBS, followed by 5 ​min washing with 1X PBS. The samples were mounted atop microscopic glass slides using 10 ​μl Prolong gold antifade reagent containing DAPI (4’,6-diamidino-2-phenylindole) (Thermo Fisher Scientific, US) and were imaged using a fluorescence microscope (ZOE™ fluorescent cell imager, Bio-Rad, The Netherlands). A similar immunocytochemical staining procedure was performed on separate samples to visualize and analyze YAP after 1 day of culture. To this end, a mouse monoclonal anti-YAP1 antibody (1:100 in 1% BSA/PBS, Santa Cruz Biotechnology, Germany) and Alexa Fluor 488, donkey anti-mouse polyclonal secondary antibody (1:200 in 1% BSA/PBS, Thermo Fisher Scientific, US) were used.

Finally, in order to evaluate the effects of surface topography on the expression of osteogenic markers in hMSCs, the cells were stained for Runx2 after 9 days of culture through incubation in recombinant anti-Runx2 rabbit monoclonal primary antibody (1:250 in 1% BSA/PBS, Abcam, UK). The cells were then incubated in Alexa Fluor 488, donkey anti-rabbit polyclonal secondary antibody (1:200 in 1% BSA/PBS, Thermo Fisher Scientific, US), before being washed and imaged as described above.

#### 2.2.3 Investigation of cell-nanopillars interface

In order to further investigate the interactions between the cells and high aspect ratio nanopillars of bTi, focused ion beam scanning electron microscopy (FIB-SEM, FEI, Helios Nano Lab 650, US) was performed to acquire tilted and cross-sectional views of the interface between the cells and nanopillars. For the SEM observations, the samples stained for vinculin and actin on day 1 of culture were dehydrated using incremental volumes of ethanol (*i.e.*, washing samples with 50%, 70%, and 96% ethanol solutions for 15 ​min, 20 ​min, and 20 ​min, respectively), air-dried, and gold-sputtered before SEM imaging. To acquire cross-sectional views, the samples were tilted to 52°, at which angle the surface was milled using Gallium ions with a 7.7 ​pA ion beam (Z ​= ​1 ​μm, operating voltage ​= ​30 ​kV).

#### 2.2.4 PrestoBlue assay

The metabolic activity of the hMSCs, seeded on the flat Ti and bTi surfaces, was measured by a PrestoBlue assay after 1, 4, and 7 days of culture. Briefly, all samples were incubated in 250 ​μl of culture medium supplemented with 25 ​μl PrestoBlue reagent (Thermo Fisher Scientific, US) for 1 ​h at 37 ​°C and 5% CO_2_. Thereafter, 100 ​μl of the supernatant from each well was transferred to a 96 well-plate (Greiner, Bio-One, The Netherlands) in duplicate. The fluorescence was measured at an excitation wavelength of 530 ​nm and an emission wavelength of 595 ​nm with a Victor X3 microplate reader (PerkinElmer, The Netherlands).

### 2.3 Inhibition studies

To study the role of FAK, ROCK, and YAP in the regulation of Runx2 as an osteogenic marker in hMSCs while exposed to high aspect ratio nanopillars, each of those factors was inhibited in separate dedicated experiments. The pre-culture, cell seeding, and the composition of the culture medium were kept identical to the procedures described in Section 2.2.1 during these experiments while the following inhibitors were added to the culture medium upon cell seeding and medium refreshing: 10 ​μM PF-573228 (FAK inhibitor, Sigma-Aldrich, Germany), 10 ​μM Y-27632 (ROCK inhibitor, Abcam, The Netherlands), and 10 ​μM Verteporfin (YAP inhibitor, Sigma-Aldrich, Germany). The short-term adaptation of the cells to the surfaces under each new condition as well as the expression of a major osteogenic marker (*i.e.*, Runx2) were evaluated through immunocytochemical staining procedures that were identical to those applied in Section 2.2.2.

### 2.4 Fluorescence image analysis

ImageJ 1.53c (NIH, US) was used to extract and quantify data from the fluorescent images. The quantification of the number and area of FAs (*i.e.*, vinculin) as well as morphological characteristics of the cell body and nucleus, such as their area and aspect ratio, under the normal or inhibition conditions, was carried out by thresholding the grayscale images of F-actin and vinculin and running the Analyze Particles command as described elsewhere [7,27]. The shape index of the cell body and nucleus was calculated as follows [28]:

$$Shape\;index=\frac{4\pi A}{P^{2}}$$

where *A* represents the cell or nucleus area and *P* is their perimeter.

The images of YAP (in the experiment with no inhibition) were also processed similarly to the FAs. Briefly, the background was subtracted using the Sliding Paraboloid option with a rolling ball radius of 50 pixels. The local contrast of the image was then enhanced by running the CLAHE plugin with a block size of 19, histogram bins of 256, and a maximum slope of 6. To further minimize the background, the mathematical exponential function (EXP) was applied. The brightness, contrast, and threshold were then automatically adjusted before measuring the stained area using the Analyze Particles command. The measured area was then normalized with respect to the area of the same cell. Moreover, for further comparisons based on the nuclear YAP intensity, the mean gray value of the expressed YAP was measured within the cell nucleus area. The signal intensity of Runx2 expressed by the cells was also measured similarly under all culture conditions.

### 2.5 Statistical analysis

For all the experiments, the raw data were first tested for normality using the D'Agostino-Pearson omnibus normality test in Prism (version 9.2.0, GraphPad, US). In the cases where the sample size was too small for such a test, the Shapiro-Wilk normality test was performed. For normally distributed datasets, the unpaired Student's *t*-test with the Welch's correction was performed to determine the statistical significance of the differences between the means of the different experimental groups. For the datasets that did not pass the normality test, the non-parametrical Mann Whitney test was employed. In addition, the results of the PrestoBlue assay were analyzed using two-way ANOVA, followed by Tukey's multiple comparisons *post hoc* analysis. All the data are presented as mean ​± ​standard deviation and a *p-*value below 0.05 was considered to indicate statistical significance.

## 3 Results

### 3.1 Characteristics of high aspect ratio bTi nanopillars

**Fig. 2:**
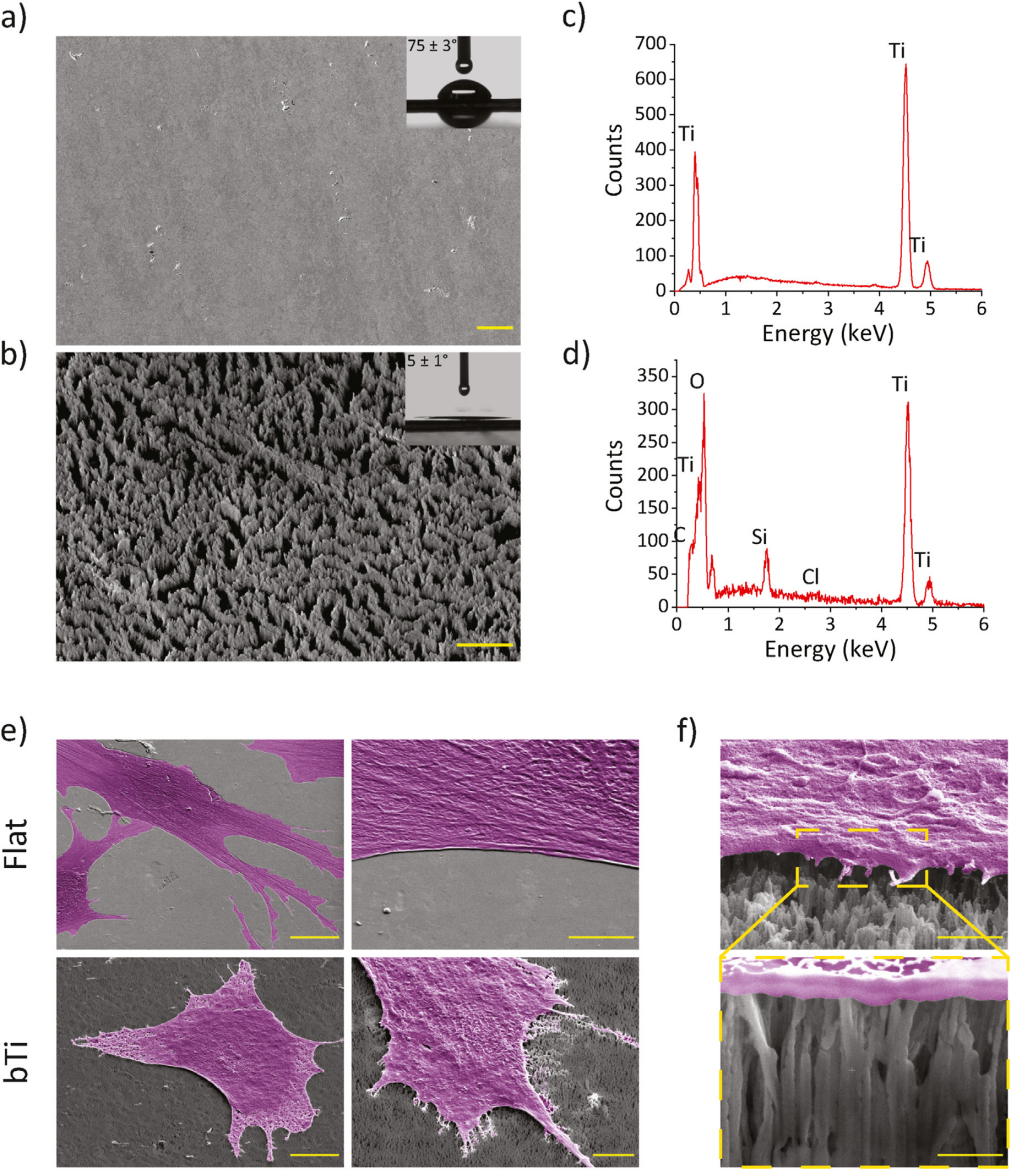
a) A top-view SEM image of the polished Ti surfaces before the application of the ICP RIE process. The inset depicts the water droplet residing on the surface after 5 ​s. Scale bar ​= ​20 ​μm. b) A tilted-view SEM image of the bTi surfaces made through ICP RIE. The inset depicts the water droplet residing on the surface after 5 ​s. Scale bar ​= ​5 ​μm. EDS spectra of c) flat Ti and d) bTi surfaces, showing the most abundant chemical elements identified on each surface. e) The representative SEM images of hMSCs settled on the flat Ti and bTi surfaces for 1 day. Left column: low magnification images showing the cell morphology. Scale bar ​= ​20 ​μm. Right column: higher magnification images showing the presence (absence) of filopodia around the cell periphery on the bTi (flat Ti) surfaces. Scale bar ​= ​5 ​μm. f) A high magnification tilted view of the cell periphery in contact with high aspect ratio nanopillars, scale bar ​= ​1 ​μm, and a cross-sectional view of the interface (shown by yellow dashed lines), scale bar ​= ​500 ​nm. The cells have been false-colored for better visualization.

The ICP RIE processing conditions used in this study resulted in the generation of pillar-shaped structures on the Ti surface (Fig. 2a and b) with a homogenous black appearance due to the light absorption within the fabricated structures, similar to the previous reports in the literature [7,13,14]. SEM observations revealed that the tips of the pillars were relatively separated from each other but the pillars were not sparsely distributed on the surface. In fact, the bTi surface seems to be comprised of partitions of pillars. Precise, objective measurement of the height of single pillars was not possible because the lowermost point of the pillars cannot be always detected (or even defined). However, the analysis of SEM images suggests a height of 700 ​nm to 1 ​μm. The diameter of the single pillar tips was <100 ​nm and the average value of aspect ratio for the pillars was approximately 12.1.

The chemical composition of the bTi surface was different from that of pure flat Ti as oxygen could be abundantly detected on its surface besides Ti (Fig. 2c and d). The ICP RIE process had also introduced slight amounts of silicon and chlorine to the surface. Furthermore, the etching process resulted in a drastic change in the wettability of the Ti surfaces as their static water contact angle decreased from 75 ​± ​3° to 5 ​± ​1° after ICP RIE processing (insets of Fig. 2a and b).

### 3.2 Effects of high aspect ratio nanopillars on the behavior of hMSCs

**Fig. 3:**
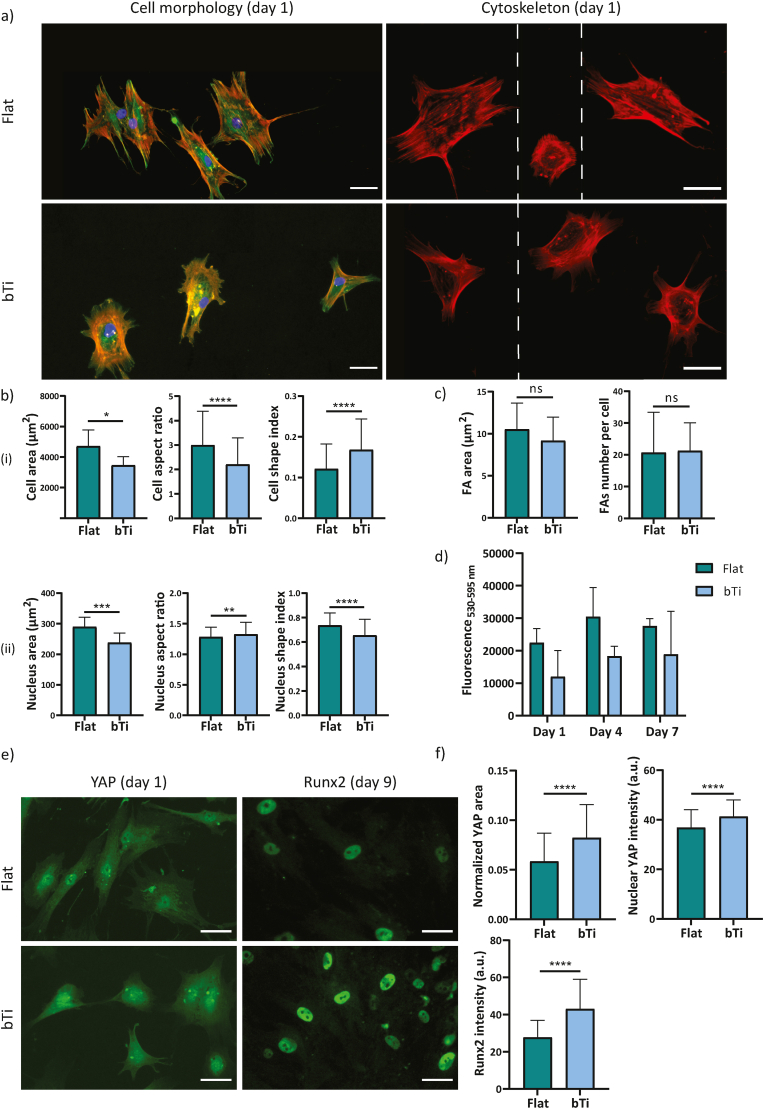
The adaptation and interactions of hMSCs with the flat Ti and bTi surfaces. a) The immunocytochemical staining of vinculin (green), actin (red), and nucleus (blue), visualizing the morphology, formation, and distribution of FAs as well as the cytoskeletal organization of the hMSCs cultured on the flat Ti and bTi surfaces for 1 day. The white dashed lines indicate a collage is made of images from different samples. Scale bar ​= ​50 ​μm. b) The morphological characteristics of the cell body (i) and nucleus (ii), including the area, aspect ratio, and shape index. At least 200 ​cells were analyzed per study group. c) The average area and number of FAs per cell measured for 15 ​cells on the flat Ti and bTi surfaces. d) The metabolic activity of the hMSCs cultured on the flat Ti and bTi surfaces, measured by the PrestoBlue assay over 7 days. e) The immunocytochemical staining of YAP and Runx2 for the hMSCs cultured on the flat Ti and bTi surfaces after 1 and 9 days, respectively. Scale bar ​= ​50 ​μm. f) The average YAP area normalized with respect to the cell area, nuclear YAP signal intensity, and Runx2 signal intensity. 100 and 50 ​cells were analyzed per study group for YAP and Runx2, respectively. ∗*p* ​< ​0.05, ∗∗*p* ​< ​0.01, ∗∗∗*p* ​< ​0.001, and ∗∗∗∗*p* ​< ​0.0001.

hMSCs maintained their fibroblast-like cell morphology when cultured on flat Ti and were observed to be well-spread on the surface already after 1 day of culture (Fig. 3a). The cells attached to the high aspect ratio bTi nanopillars possessed a significantly smaller area as compared to the cells attached to the flat Ti surfaces (Fig. 3b). Moreover, they had a lower (higher) aspect ratio (shape index) as compared to the cells cultured on flat Ti, indicating a less elongated (more rounded) morphology (Fig. 3b). The evaluation of the morphology of the cell nuclei revealed an opposite trend. While the cell nucleus area was found to be smaller on the bTi samples, the aspect ratio (shape index) of the nucleus was higher (lower) on those surfaces (Fig. 3b). On the flat Ti surfaces, the actin fibers of the cells were mostly oriented along the major axis of the cell body. In contrast, a uniform organization of actin fibers could not be identified in the cells cultured on the bTi surfaces where the actin fibers appeared to be thicker on the cell periphery, surrounding the more internal areas of the cell body (*e.g.*, cell nuclear area) (Fig. 3a). Additionally, the cells formed FAs on both types of surfaces. On the bTi surfaces, they were mostly present on the cell periphery where filopodia had been also formed (Fig. 3a). The average area of FAs and their number per cell were not significantly affected by the bTi nanopillars (Fig. 3c).

The FIB-SEM images confirmed that the hMSCs form abundant filopodia around their periphery upon attachment to the bTi nanopillars. This was not observed in the cells residing on the flat Ti surfaces (Fig. 2e). The cells had settled on the bTi nanopillars with a top state meaning that the whole cell body was spread on top of the nanopillars. Furthermore, we did not observe any signs of the deep penetration of the nanopillars into the cell body (*i.e.*, any ruptures or any signs of engulfment by the cell membrane) or local cell stretching in between the adjacent nanopillars (Fig. 2f).

The results of the PrestoBlue assay showed that both types of surfaces can support the survival and proliferation of hMSCs and that there was no significant difference between the overall metabolic activity of the cells on the flat Ti and bTi surfaces within a time span of 1 week (Fig. 3d).

The hMSCs expressed YAP and Runx2 on days 1 and 9 of culture, respectively, regardless of the surface they were residing on (Fig. 3e). However, significantly larger areas were stained for YAP in the cells residing on the bTi surfaces (when normalized to the actual cell area) (Fig. 3f). In addition, while the nuclear YAP percentage was significantly higher in the cells residing on flat Ti surfaces, the signal intensity of YAP within both the cell nuclear area and cytoplasmic area was significantly higher on the bTi surfaces (Fig. 3f and S7), indicating a higher presence of YAP in the nucleus. Similarly, the signal intensity of Runx2 expressed by the cells residing on the bTi surfaces was significantly higher than on flat Ti (Fig. 3f). Supplementary examination of surface mineralization by cells also indicated significantly enhanced matrix mineralization on the bTi surfaces as compared to flat Ti (Fig. S6), which is in line with the results of previous reports that cultured MC3T3-E1 preosteoblasts and hASCs on similar surfaces [7,15].

### 3.3 Effects of FAK inhibition on hMSCs

**Fig. 4:**
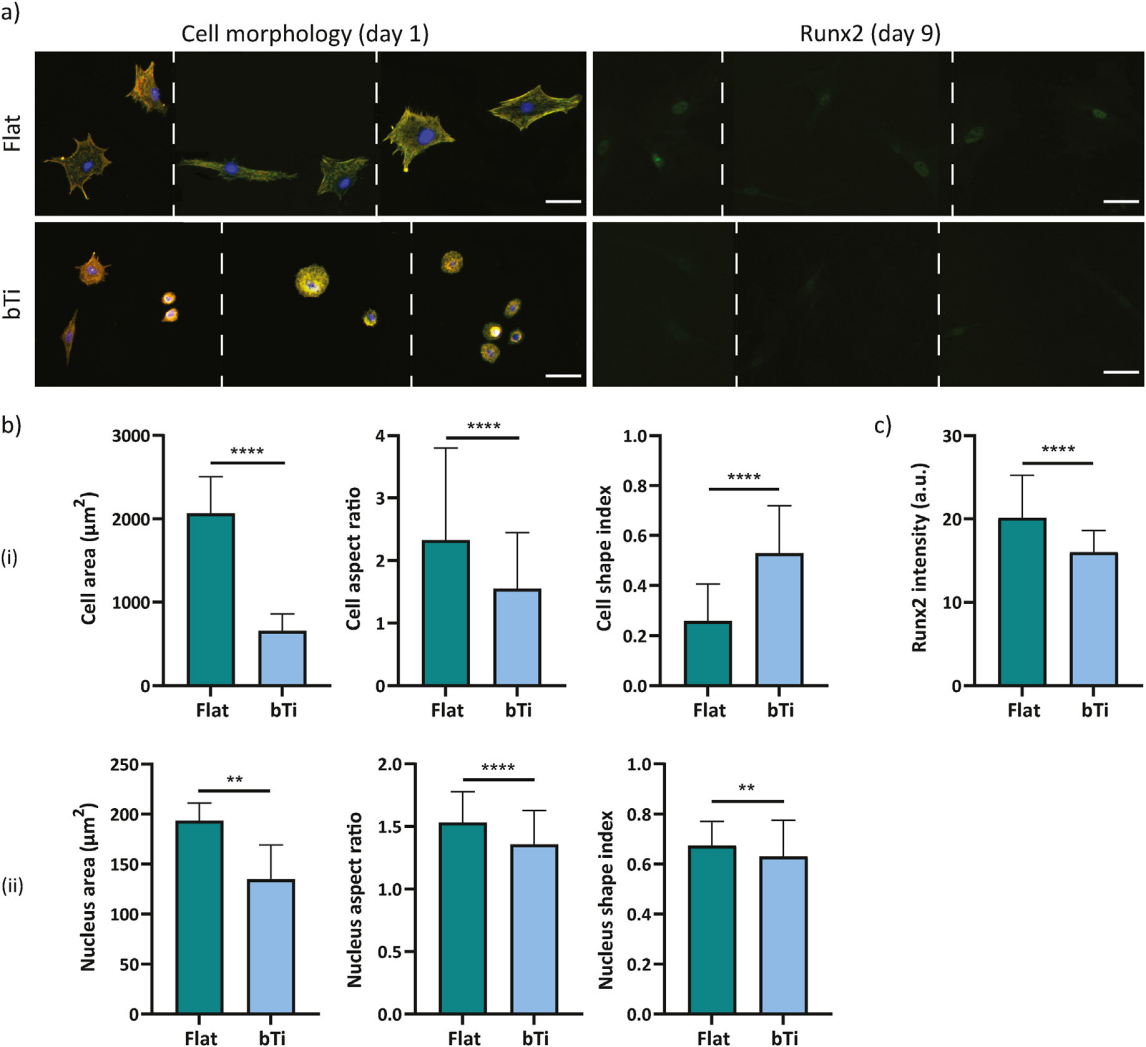
The adaptation and interactions of the hMSCs with the flat Ti and bTi surfaces after the inhibition of FAK. a) The morphology of hMSCs and expression of Runx2 after 1 and 9 days of culture, respectively, on the flat Ti and bTi surfaces. The white dashed lines indicate that a collage is made of images from different samples. Scale bar ​= ​50 ​μm. b) The morphological characteristics of the cell body (i) and nucleus (ii), including the area, aspect ratio, and shape index. At least 200 ​cells were analyzed per study group. c) The intensity of the Runx2 signal for the hMSCs residing on the flat Ti and bTi surfaces. At least 50 ​cells were analyzed per study group. ∗∗*p* ​< ​0.01 and ∗∗∗∗*p* ​< ​0.0001.

The inhibition of FAK drastically affected the morphological characteristics of the hMSCs on both flat and bTi surfaces. After 1 day of culture, the cells still had a polygonal shape on flat Ti while their shape had changed to an extremely rounded state on the bTi samples (Fig. 4a). On both types of surfaces, the cells had a significantly smaller area as compared to the no-inhibition conditions. In fact, the average area of cells on the flat and bTi samples under the normal condition dropped from around 4700 and 3500 ​μm^2^, respectively, to around 2000 and 650 ​μm^2^, respectively, upon FAK inhibition (Fig. 3, Fig. 4b). The values of the cell aspect ratio and shape index also confirmed the significant roundness of the cell morphology on bTi as compared to the flat Ti (Fig. 4b). The changes were not only limited to the cell body but there was also a significant decrease in the cell nucleus area and elongation on the bTi samples as compared to the flat Ti (Fig. 4b).

Furthermore, the immunocytochemical staining of Runx2 revealed that the inhibition of FAK downregulates the expression of Runx2 as the signal intensity significantly decreased in comparison with the normal conditions (Fig. 3, Fig. 4a,c).

### 3.4 Effects of ROCK inhibition on hMSCs

**Fig. 5:**
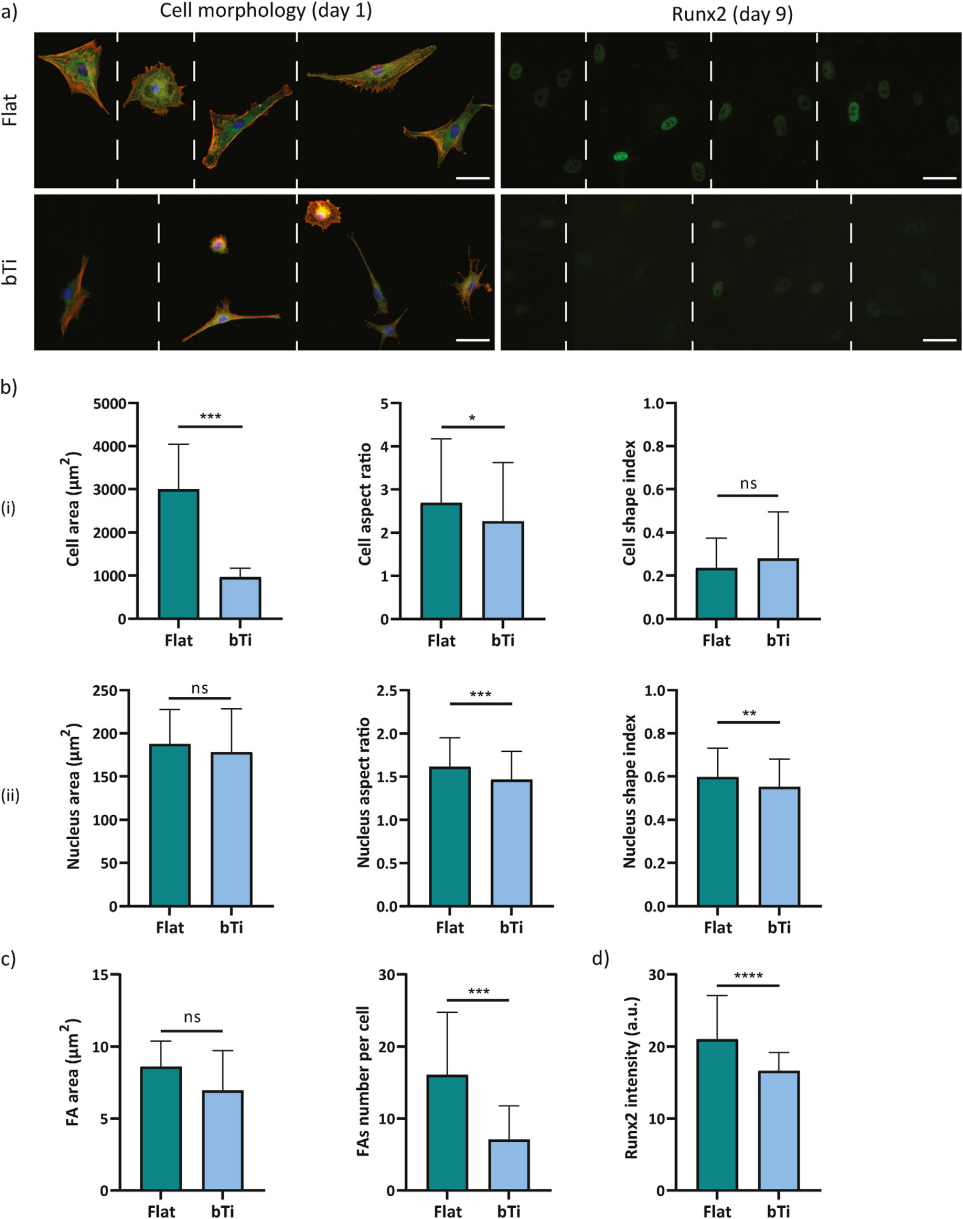
The adaptation and interactions of the hMSCs with the flat Ti and bTi surfaces after the inhibition of ROCK. a) The morphology of the hMSCs and expression of Runx2 after 1 and 9 days of culture, respectively, on the flat Ti and bTi surfaces. The white dashed lines indicate that a collage is made of images from different samples. Scale bar ​= ​50 ​μm. b) The morphological characteristics of the cell body (i) and nucleus (ii), including the area, aspect ratio, and shape index. At least 100 ​cells were analyzed per study group. c) The average area and number of FAs per cell were measured for 15 ​cells on the flat Ti and bTi surfaces. d) The intensity of the Runx2 signal for the hMSCs residing on the flat Ti and bTi surfaces. At least 200 ​cells were analyzed per study group. ∗*p* ​< ​0.05, ∗∗*p* ​< ​0.01, ∗∗∗*p* ​< ​0.001, and ∗∗∗∗*p* ​< ​0.0001.

The inhibition of ROCK did not induce an apparent change in the polygonal morphology of the hMSCs on flat Ti but resulted in different morphologies displayed by the cells residing on the bTi surfaces, for instance stellate, polarized, and round shapes (Fig. 5a). The average cell area was also significantly lower on the bTi samples as compared to the flat Ti (around 950 ​μm^2^ as compared to around 3000 ​μm^2^) (Fig. 5b). Although the cells residing on the flat Ti had a higher aspect ratio than on the bTi samples, the differences in the cell shape index were insignificant, most likely due to the various cell shapes found on the bTi surfaces (Fig. 5b). In addition, the presence of bTi nanopillars did not significantly affect the cell nucleus area. However, the nucleus was less elongated on these samples as compared to the flat Ti surfaces (Fig. 5b). Similar to the normal conditions, the average focal adhesion area was not significantly different on the flat Ti and bTi surfaces (Fig. 3, Fig. 5c). But, the cells residing on the bTi samples had formed fewer FAs around their periphery (Fig. 3, Fig. 5c).

The expression of Runx2 was downregulated after ROCK inhibition on both the flat Ti and bTi samples (Fig. 3, Fig. 5a). Similar to the effects of FAK inhibition, the inhibition of ROCK was associated with a more remarkable decrease in the signal intensity of Runx2 in the cells residing on the bTi surfaces (Fig. 3, Fig. 5d).

### 3.5 Effects of YAP inhibition on hMSCs

**Fig. 6:**
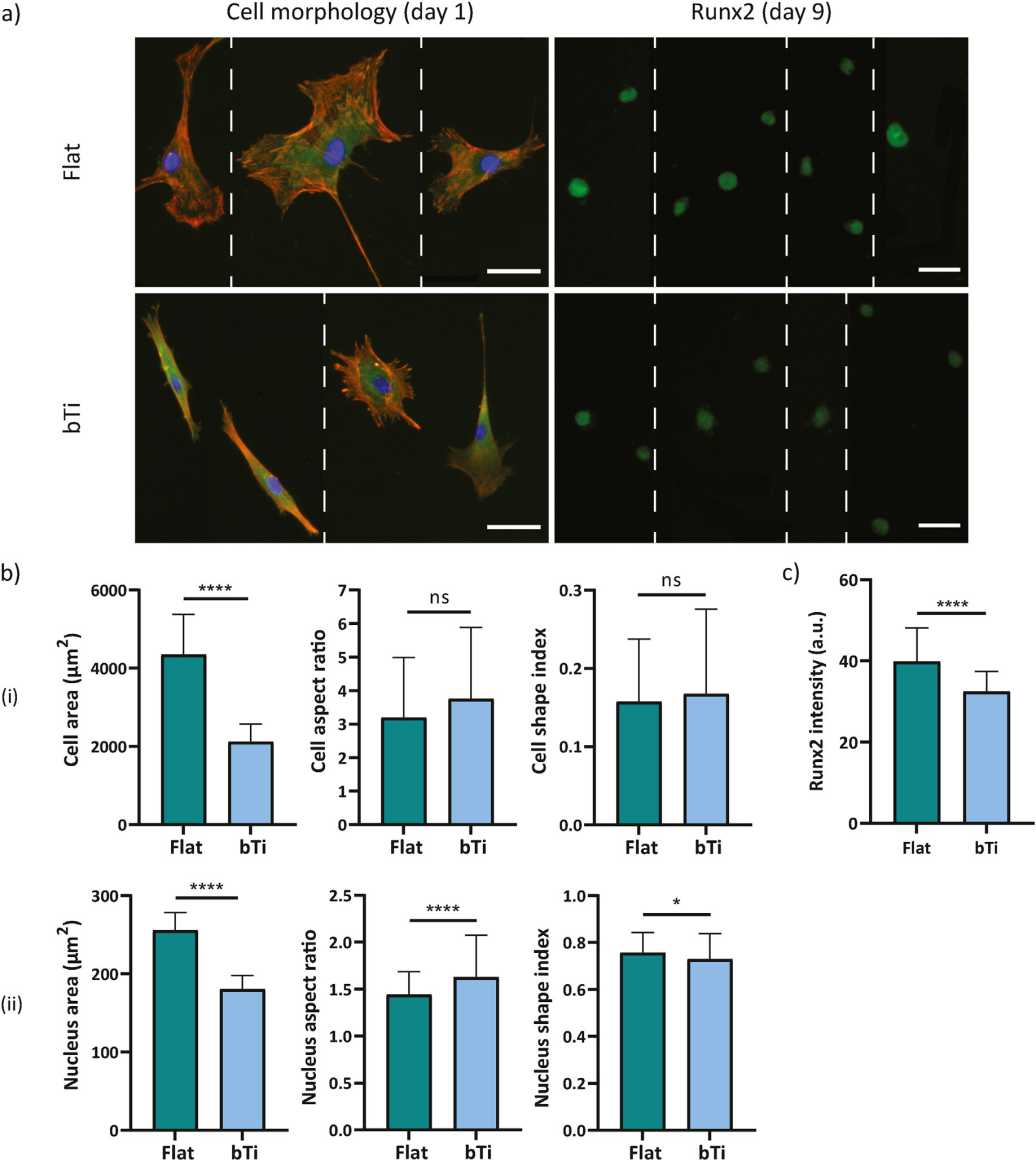
The adaptation and interactions of the hMSCs with the flat Ti and bTi surfaces after the inhibition of YAP. a) The morphology of the hMSCs and expression of Runx2 after 1 and 9 days of culture, respectively, on the flat Ti and bTi surfaces. The white dashed lines indicate that a collage is made of images from different samples. Scale bar ​= ​100 ​μm. b) The morphological characteristics of the cell body (i) and nucleus (ii), including the area, aspect ratio, and shape index. At least 100 ​cells were analyzed per study group. c) The intensity of the Runx2 signal for the hMSCs residing on the flat Ti and bTi surfaces. At least 50 ​cells were analyzed per study group. ∗*p* ​< ​0.05 and ∗∗∗∗*p* ​< ​0.0001.

The YAP-inhibited hMSCs cultured on the flat Ti samples did not show a very different morphology as compared to the cells not exposed to any inhibitory agent (Fig. 3, Fig. 6a). These cells maintained a polygonal shape and their area was not significantly affected (*i.e.*, an average area of around 4300 ​μm^2^ as compared to around 4700 ​μm^2^ under the normal conditions) (Fig. 3, Fig. 6b). However, the cells residing on the bTi surfaces displayed an altered morphology (*i.e.*, both stellate and highly elongated morphologies) (Fig. 6a) and a significantly decreased spreading area (*i.e.*, 2100 ​μm^2^) (Fig. 6b). Despite the differences in the cell area, the cell aspect ratio and shape index were not significantly different between the flat Ti and bTi surfaces (Fig. 6b). Furthermore, similar to the normal conditions, the cells residing on the bTi surfaces had smaller but less rounded and more elongated nuclei as compared to the cells attached to the flat Ti surfaces (Fig. 3, Fig. 6b).

Unlike the previous inhibition experiments, the YAP inhibition experiments increased the intensity of the Runx2 signal for the cells residing on the flat Ti surfaces (Fig. 4, Fig. 5, Fig. 6c). The bTi surfaces significantly reduced the intensity of the Runx2 signal but the intensity values were still much higher than in the FAK and ROCK inhibition experiments (Fig. 4, Fig. 5, Fig. 6c).

## 4 Discussion

The emergence of high aspect ratio nanostructured surfaces has opened a new horizon in the design and manufacturing of cell-instructive biomaterials. These surfaces provide the cells with extreme topographical cues and alter their response to the extracellular microenvironment. The huge potential of these surfaces to influence the mechanosensing pathways of cells and the countless scenarios in which the cells undergo different differentiation processes have underscored the importance of in-depth studies on the function of such biological interfaces [8]. Previous studies have shown the effectiveness of (ICP) RIE in the production of bactericidal and osteogenic high aspect ratio nanopillars on Ti as the most clinically relevant material for orthopedic applications [7,[13], [14], [15]]. The existing literature consistently shows that high aspect ratio bTi nanopillars affect the cell morphology, spreading area, and adhesion complexes in both primary cells [13,15] and cell lines [7]. However, the pathways involved in the transduction of mechanical forces, the interpretation of the cell shape, and, eventually, the transcription of osteogenic markers in the cell nucleus are not yet fully elucidated. In the current study, we focused on the interactions of hMSCs with high aspect ratio bTi nanopillars and the role of FAK (as an important initiator-regulator of FA formation and maturation), ROCK (as a regulator of cell contractility), and YAP (as a nuclear transcriptional regulator) in the expression of osteogenic markers induced by the nanopillars.

While the high aspect ratio nanopillars did not impair the attachment, survival, and metabolic activity of the cells, they gave rise to a cell morphology that was different from the one observed on the polished flat surfaces. The extreme change in the surface topography provides the cells with a novel landscape of potential anchorage points. The initially (*i.e.*, day 1) confined cell area on the nanopillars as compared to the flat surface together with the development of abundant filopodia indicates that the cells are still probing the surface to establish more stable attachments by finding more suitable ligands for integrin binding, and an optimized membrane trafficking, resulting in a slower adaptation to the surface [7,[29], [30], [31]]. It is noteworthy that the discrepancies in the metabolic activity of cells residing on flat Ti and bTi surfaces (although statistically insignificant) require further investigation in future studies to elucidate the relationship between cell mechanics, cell shape, and metabolism [32]. It may be also investigated whether the bTi nanopillars affect the molecular diffusion via cell membrane that has been shown in recent studies on cell-nanotopography interactions [33]. The altered cell morphology was also associated with differences in the organization of the cytoskeleton and the formation of FAs. Although the cells attached to the bTi nanopillars had, on average, a lower aspect ratio as compared to the cells residing on the flat Ti, they showed more cellular extensions. Developing these cell protrusions in different directions led to a higher cell shape index (*i.e.*, the perimeter closing in on the area). Interestingly, the poorly-organized F-actin can be mostly observed to be oriented in the direction of these protrusions. The observed cytoskeleton organization in these cells could imply a contractile spreading phase [34], which is in line with the previous points regarding the slower adaptation to the bTi surface. Moreover, an anisotropic distribution of FAs was observed on the bTi surface, as they were predominantly formed at those locally elongated cell edges. It is difficult to determine whether these FAs are merely nascent adhesions or long-lasting stable ones. On one hand, they are not completely co-localized with underdeveloped F-actin while, on the other hand, they are oval-shaped rather than round-shaped and have an average area that is comparable to the FAs formed on flat Ti. Further analysis of integrin clustering [35,36] or the application of super-resolution microscopy and fluorescence resonance energy transfer (FRET) microscopy to detect the upward shifting of vinculin away from the cell membrane [37] could clarify these contradictory findings regarding the maturation of FAs on high aspect ratio nanopillars. Here, we may conclude that there are already sufficient forces generated via actomyosin contractility, resulting in the activation and recruitment of vinculin to the adhesion sites [4,38]. Another point to consider is that the morphology and dynamics of cell nuclei are influenced by the forces transduced through FAs [39,40]. For instance, unevenly distributed mature FAs could stretch the cell nucleus, thereby enhancing the osteogenic differentiation of cells [40]. Our findings showed that the cell nucleus is more elongated and less rounded on the bTi surfaces as compared to flat Ti, which might be linked to the different distribution of FAs.

Even though the hMSCs required more time to adapt to the bTi surfaces, they exhibited a higher intensity of the YAP signal in their nuclei and had a larger total cytoplasmic/nuclear YAP area. It is likely that the YAP nuclear translocation has been continuing to an even greater extent after this time point. Several studies have reported a connection between nuclear translocation of YAP and enhanced osteogenic differentiation [[41], [42], [43], [44]]. Particularly, an incremental nuclear translocation of YAP and Runx2 has been previously shown in hMSCs undergoing osteogenic differentiation induced by altered ECM ligand spacing [45] or cyclic stretch [46]. In contrast, some studies on the osteogenic properties of TiO_2_ nanotubes [47,48] and electrospun nanofibers [22] suggest that dissociation of nuclear YAP/Runx2 complexes and the expelling of YAP to the cytoplasm enable Runx2 to induce skeletal gene expression. Our results indicate that in the case of high aspect ratio bTi nanopillars, a correlation exists between the nuclear translocation of YAP and the expression of Runx2.

To further investigate the mechanisms underlying these observations, we investigated the effects of inhibiting FAK as a substantial part of the integrin signaling layer of FAs [49] which is crucial for the initiation of outside-in force transduction. The inhibition of FAK resulted in a decreased cell spreading area on both bTi and flat Ti surfaces, possibly due to the inability of the cells to secure strong adhesions on the surface. The inhibition was also correlated with a significantly decreased Runx2 signal intensity, which might be directly related to the disruption of FAs in the sense that force transduction from the integrins to the nucleus is hindered [50]. The reduction in the Runx2 intensity was more severe in the cells residing on the bTi nanopillars. The extremely small and rounded morphology of these cells has most likely limited the cytoskeleton organization. In addition, the disappearance of the heterogeneous distribution of FAs on the bTi surface may have reduced the elongation of the nuclei as compared to the cells residing on the flat Ti surface, reinforcing the hypothesis that, unlike the normal condition, here the nuclei are undertensioned [48,51]. Measuring the adhesion forces [52] in future research would quantitatively confirm/reject this hypothesis.

Another intracellular pathway that plays a major role in perceiving the ECM mechanics is the RhoA/ROCK pathway. RhoA which is a member of the Rho family of GTPases regulates the activity of myosin II through its effector ROCK. Through the phosphorylation of the myosin light chain (MLC), ROCK increases the contractile force generated by myosin II on actin fibers. ROCK is, thus, considered as a massive regulator of cell-generated forces [53], which consequently affects the cell shape [54,55] and the activation of FAK in an inside-out signaling process [56]. In this study, the effects of ROCK inhibition on the cell area and cell nucleus elongation and shape index were similar to those of FAK inhibition. The cell area was reduced on both flat Ti and bTi surfaces, but bTi induced a more drastic change. As explained earlier, less tensions seemed to have been exerted on the cell nucleus. Moreover, the ROCK-inhibited hMSCs showed very irregular morphologies on the bTi nanopillars, indicating the significant dependency of the cells on ROCK to display a fibroblast-like morphology when interacting with extreme topographies. The spindle-like stellar morphology has been previously observed in ROCK-inhibited hASCs [20]. Our results are also in agreement with the results of the previous studies in which the cell area decreased upon ROCK inhibition [22,57]. In addition to the cell area, ROCK inhibition has been shown to decrease the formation of FAs and their size on micropatterned surfaces [57]. Here, we did not observe a significant difference in the FA area between the flat Ti and bTi surfaces. However, the cells residing on the bTi surfaces formed significantly lower numbers of FAs. We may conclude that the higher dependency of the bTi-dwelling cells on ROCK for regulating their shape, adhesion to the surface, and endogenous forces, results in the lower expression of Runx2 in comparison to the flat Ti-dwelling cells. Such a correlation between inhibiting ROCK and the suppression of osteogenic differentiation has been previously shown in the presence [22] and absence [20] of nanostructured biomaterials.

An increased cytoskeletal tension regulated by the RhoA/ROCK pathway is believed to cause the nuclear localization of YAP [23,58]. YAP shuttling between the cytoplasm and nucleus has been shown to be controlled by the cell area [25]. Furthermore, it acts as a necessary downstream of RhoA/ROCK to regulate the formation of FAs by influencing the expression of FA-related genes [25]. However, the role of YAP in mechanotransduction cannot be considered as being merely a downstream of other pathways as YAP can also regulate its upstream factors in a feedback loop [24,59]. Based on these known facts about YAP, we expected no significant changes in the cell area after the YAP inhibition. Surprisingly, it was only true for the flat Ti surfaces and the cell area and morphology were significantly affected on the bTi nanopillars. On the flat Ti surface, the YAP inhibition was also correlated with increased Runx2 intensity as compared to the normal conditions while the Runx2 intensity significantly decreased on the bTi surfaces. Although it is not clear what exact connections exist between YAP and the regulation of the cell area and morphology on the bTi surfaces, our results suggest that the relationship between the YAP nuclear localization and the expression of Runx2 is mutually exclusive in the case of the flat Ti and bTi surfaces. Moreover, any forces exerted to the nucleus are likely regulated by the adhesion and contractility of the cells, as there was no altered trend in the morphological characteristics of the cell nuclei after YAP inhibition as compared to the normal conditions. It has been argued that the physical deformation of the nucleus through the LINC complex mediates the nuclear translocation of YAP [60]. A more recent study suggests that the nuclear translocation of YAP requires the opening of the nuclear pore complex as a result of the physical stretching of the nucleus membrane [61]. Furthermore, Lamin A/C plays a crucial role in resisting any nuclear deformations and contributing to nuclear stability and mechanics [62]. We did not observe a severe penetration of the cell body by high aspect ratio bTi nanopillars, nor did the cells engulf the nanopillars in this study. These different methods of interfacing, commonly observed in the high aspect ratio nanostructured materials, have been suggested to initiate other events such as direct nuclear mechanotransduction and endocytosis [8]. Further analysis of the nuclear mechanics and its connection to cell adhesion and contractility in future studies may clarify how YAP nuclear translocation upregulates the expression of osteogenic markers.

**Fig. 7:**
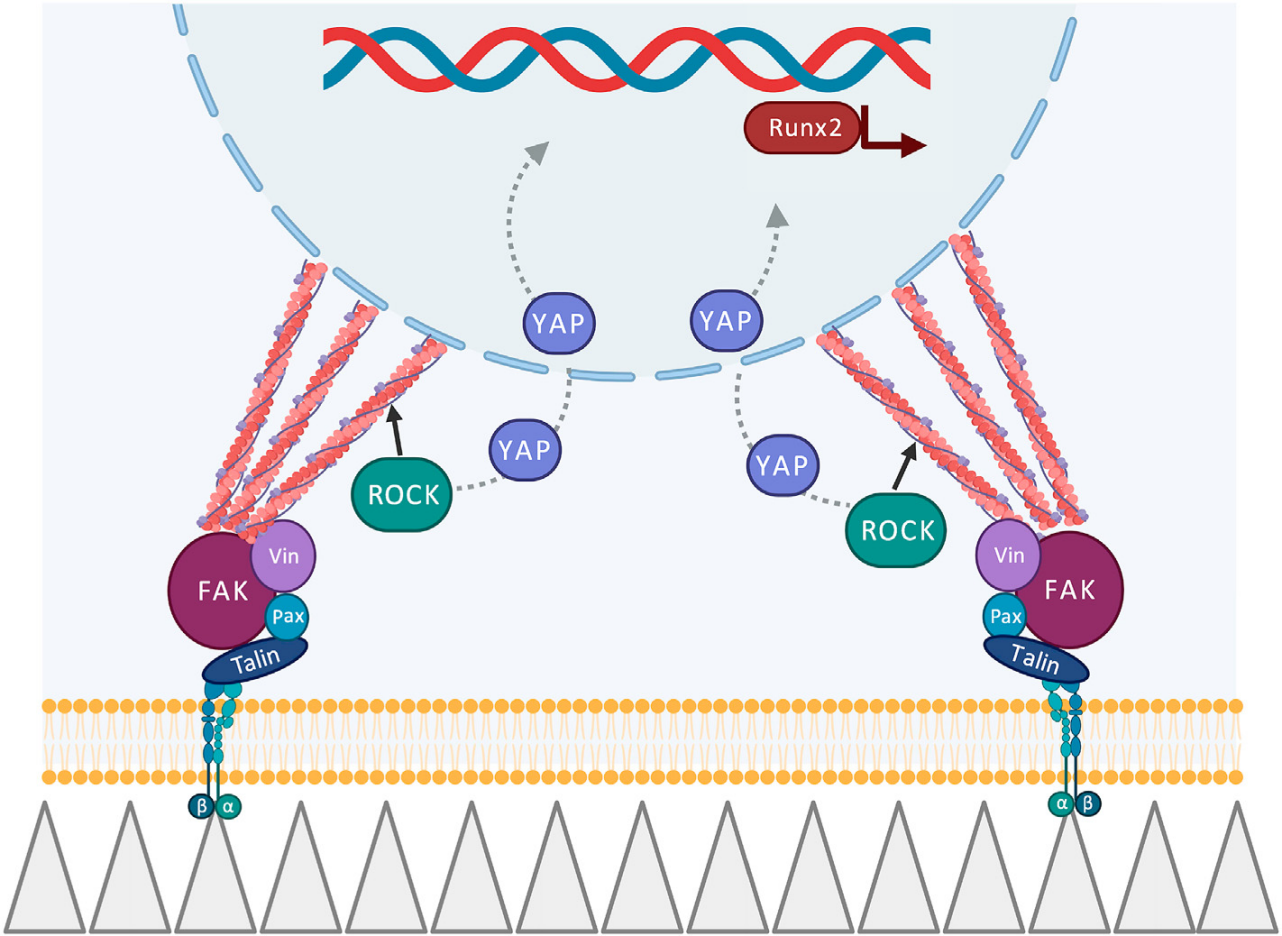
A schematic illustration of conceivable mechanotransduction pathways triggered by high aspect ratio bTi nanopillars in hMSCs. Cells largely depend on the activity of FAK and ROCK to maintain their normal shape and area on bTi nanopillars. Whether through a direct pathway via FAs and cytoskeleton or through an indirect one by enhancing nuclear translocation of YAP, hMSCs may be directed toward osteogenic differentiation indicated by enhanced expression of Runx2 and mineralization.

In summary, our findings indicate that high aspect ratio bTi nanopillars have a remarkable potential to direct hMSCs towards osteogenic lineage commitment by manipulating cell adhesion as regulated by FAK, cell contractility as regulated by ROCK, and the nuclear translocation of transcriptional factors (*e.g.*, YAP). Furthermore, these factors are interrelated and can modulate each other's roles. In other words, an osteogenic mechanotransduction pathway can be postulated in which the adhesion and adaptation of the cells to the extreme topography of the surface is highly dependent on FAK which works in close liaison with ROCK to increase the endogenous forces within the cells and determines the cell shape and area. Whether directly transducing the forces to the nucleus or indirectly enhancing the nuclear translocation of YAP, mechanical stimuli can be translated to biochemical factors, such as Runx2 in the nucleus (Fig. 7). Recent advances in the available manufacturing techniques are expected to facilitate the fabrication of 3D implantable structures out of 2D nanopatterned sheets [63,64]. The availability of such 3D implantable structures would make *in vivo* studies possible in the future to further investigate similar hypotheses and evaluate the ultimate potential of such meta-biomaterials for bone tissue regeneration.

## 5 Conclusions

High aspect ratio nanopillars created through reactive ion etching of the Ti surface hold tremendous potential for selective behavior against mammalian and bacterial cells. The rare combination of osteogenic and bactericidal properties of bTi places it in the category of cell-instructive meta-biomaterials. Here, we showed that a specific configuration of bTi nanopillars induces an increased level of Runx2 expression in hMSCs. The adaptation of the hMSCs to this surface was also associated with significant changes in the cell morphology, cytoskeleton organization, and nuclear translocation of YAP. Further investigations revealed that these phenomena are highly dependent on FAK and ROCK as the master regulators of cell adhesion and contractility, which eventually determine the cell shape and area. The mechanotransduction pathway involving FAK and ROCK either directly or indirectly (by enhancing nuclear translocation of YAP) affects the transduction of mechanical stimuli by the cell nucleus and results in the upregulation of Runx2 as a major osteogenic marker. Understanding the action mechanism of bTi nanopillars would contribute to the rational design of cell-instructive biomaterials with the aim of directing stem cells’ fate towards specific lineages.

## Credit author statement

**Khashayar Modaresifar:** Conceptualization, Methodology, Investigation, Formal analysis, Visualization, Writing-Original draft. **Mahya Ganjian:** Methodology, Investigation, Writing-Review & Editing. **Pedro J. Díaz-Payno:** Methodology, Investigation, Writing-Review & Editing. **Maria Klimopoulou:** Methodology, Writing-Review & Editing. **Marijke Koedam:** Methodology, Writing-Review & Editing. **Bram C. J. van der Eerden:** Methodology, Resources, Writing-Review & Editing. **Lidy E. Fratila-Apachitei:** Conceptualization, Resources, Writing-Review & Editing, Supervision. **Amir A. Zadpoor:** Conceptualization, Resources, Writing-Review & Editing, Supervision, Funding acquisition.

## Declaration of competing interest

The authors declare that they have no known competing financial interests or personal relationships that could have appeared to influence the work reported in this paper.

## Data Availability

Data will be made available on request.
