# Supplementary material for: Mechanotransduction in high aspect ratio nanostructured meta-biomaterials: The role of cell adhesion, contractility, and transcriptional factors
Source: Mater Today Bio. 2022 Oct 3;16:100448. doi: 10.1016/j.mtbio.2022.100448 (PMC9552121; doi:10.1016/j.mtbio.2022.100448)
Supplement: Multimedia component 1 [file mmc1.docx]

Supplementary information

Mechanotransduction in high aspect ratio nanostructured meta-biomaterials: the role of cell adhesion, contractility, and transcriptional factors

Khashayar Modaresifar^1*^, Mahya Ganjian^1^, Pedro J. Díaz-Payno^1,2^, Maria Klimopoulou^1^, Marijke Koedam^3^, Bram C. J. van der Eerden^3^, Lidy E. Fratila-Apachitei^1^, Amir A. Zadpoor^1^

*^1^ Department of Biomechanical Engineering, Faculty of Mechanical, Maritime, and Materials Engineering, Delft University of Technology, Mekelweg 2, 2628CD, Delft, The Netherlands*

*^2^Department of Orthopedics and Sports Medicine, Erasmus MC University Medical Center, Doctor Molewaterplein 40, 3015GD, Rotterdam, The Netherlands*

*^3^ Department of Internal Medicine, Erasmus MC University Medical Center, Doctor Molewaterplein 40, 3015GD, Rotterdam, The Netherlands*

* Corresponding author, email: [k.modaresifar@tudelft.nl](mailto:k.modaresifar@tudelft.nl)

# Part I: Supplementary fluorescence images


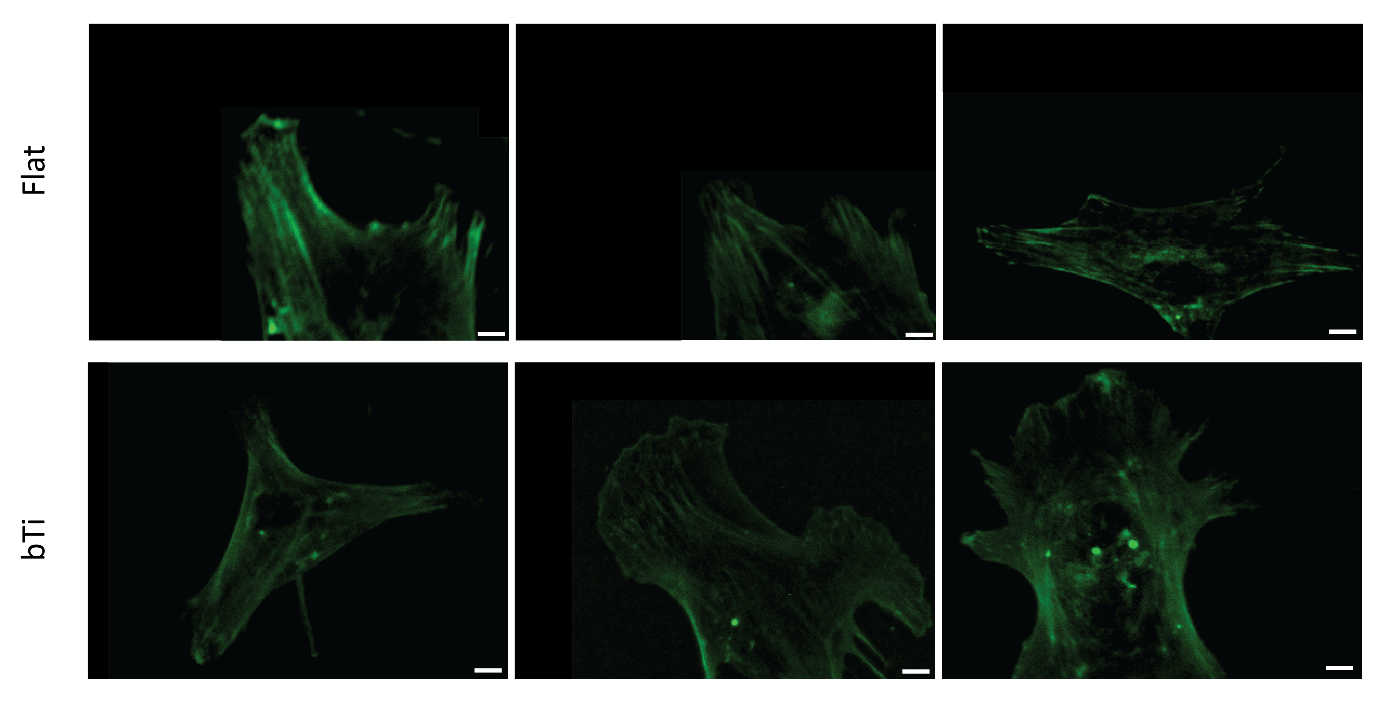


**Figure S1.** The zoomed-in images of FAs (vinculin) distribution for the hMSCs cultured on the flat Ti and bTi surfaces for 1 day. Scale bar = 10 µm.


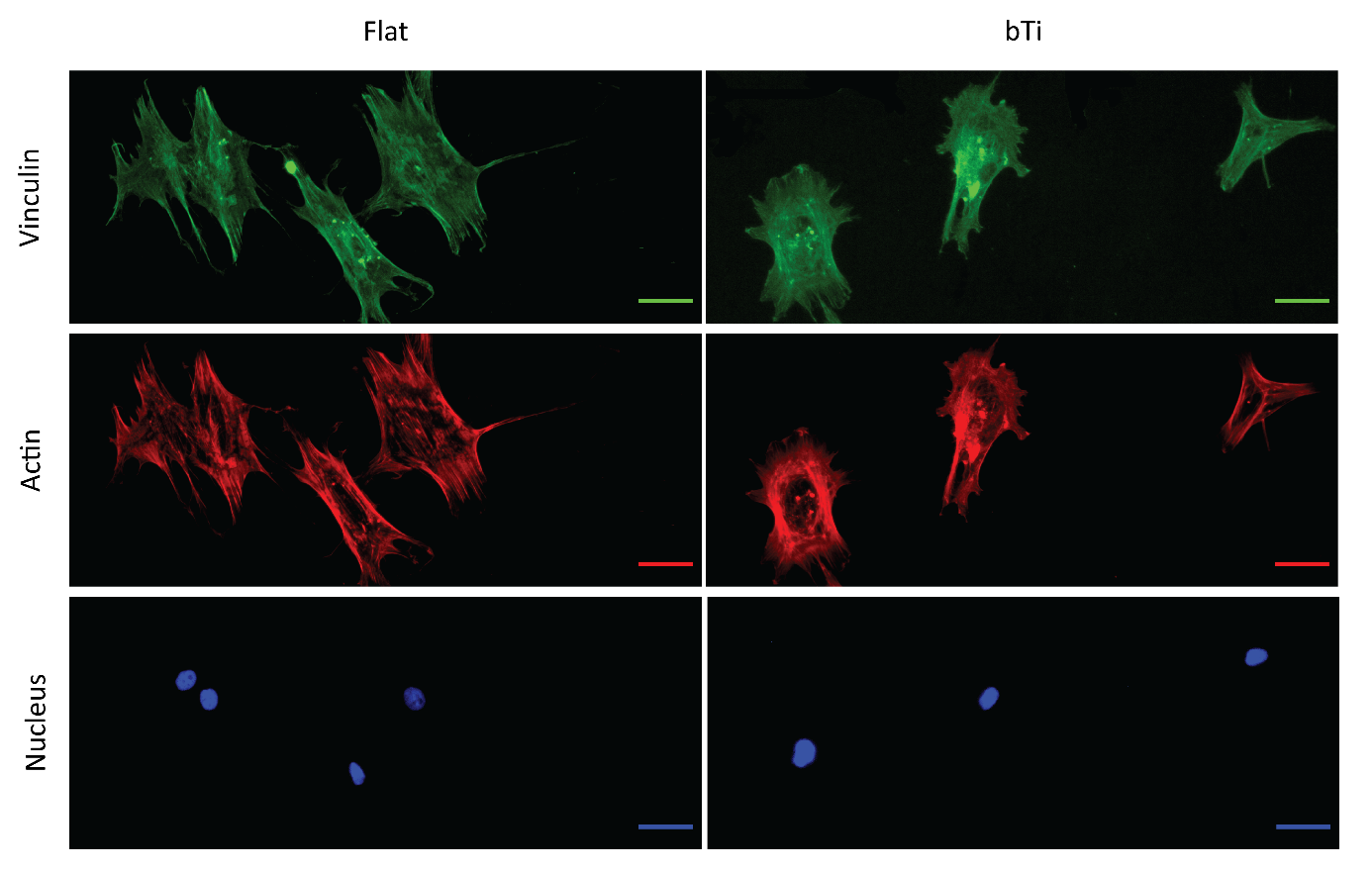


**Figure S2.** The separated channels of fluorescence the images of the hMSCs cultured on the flat Ti and bTi surfaces for 1 day. Scale bar = 50 µm.


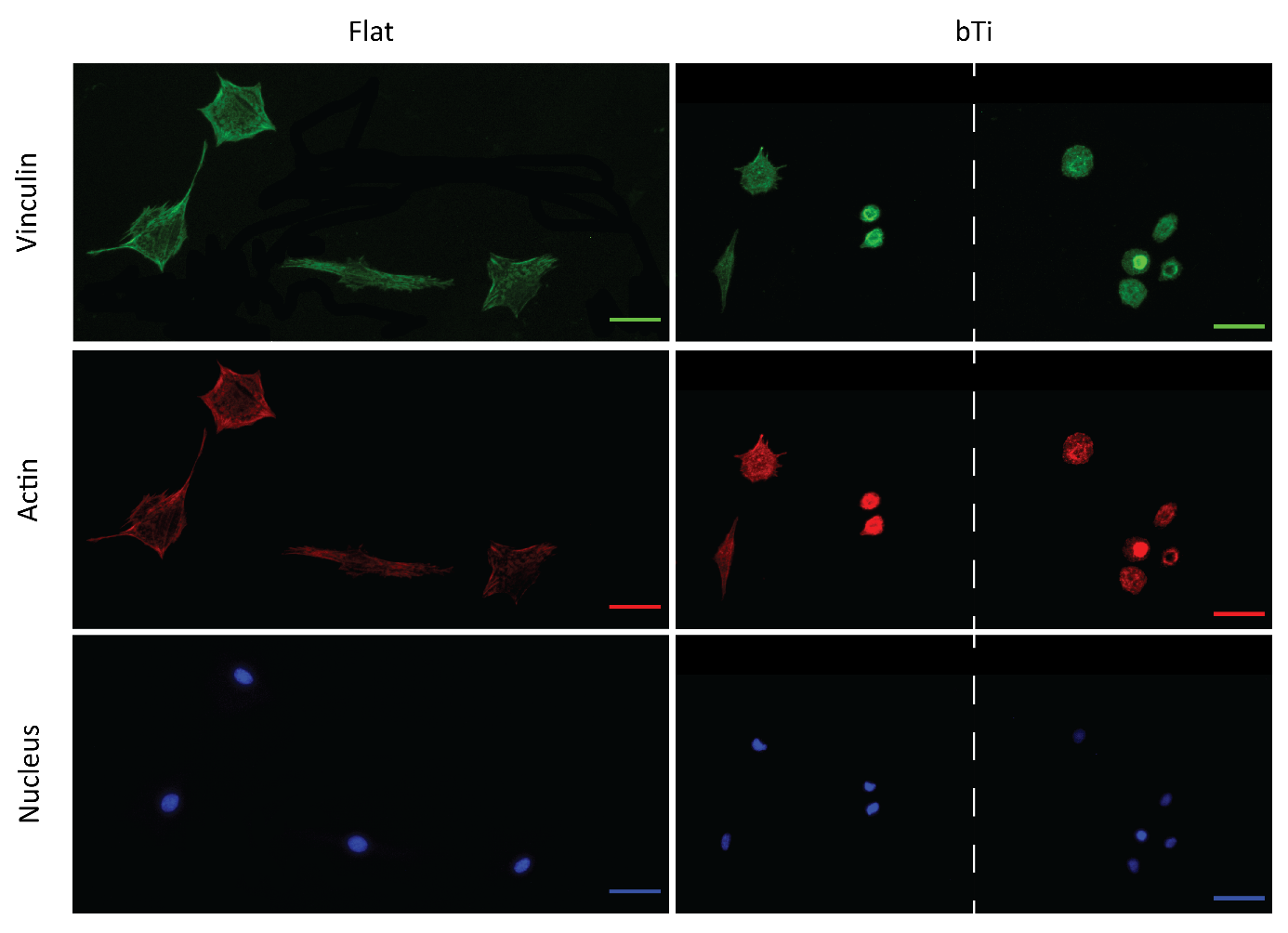


**Figure S3.** The separated channels of the fluorescence images of the FAK-inhibited hMSCs cultured on flat Ti and bTi surfaces for 1 day. Scale bar = 50 µm.


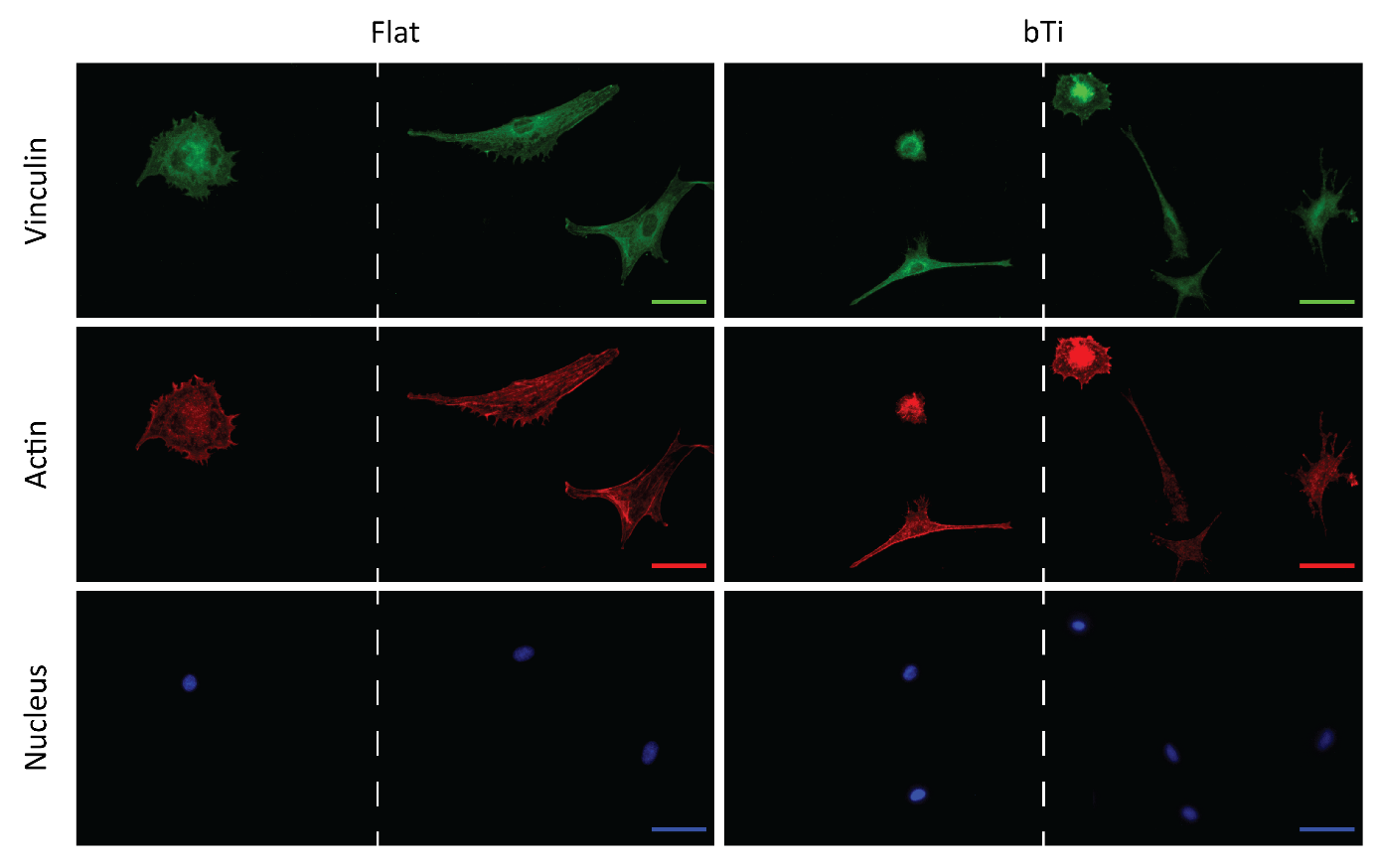


**Figure S4.** The separated channels of the fluorescence images of the ROCK-inhibited hMSCs cultured on the flat Ti and bTi surfaces for 1 day. Scale bar = 50 µm.


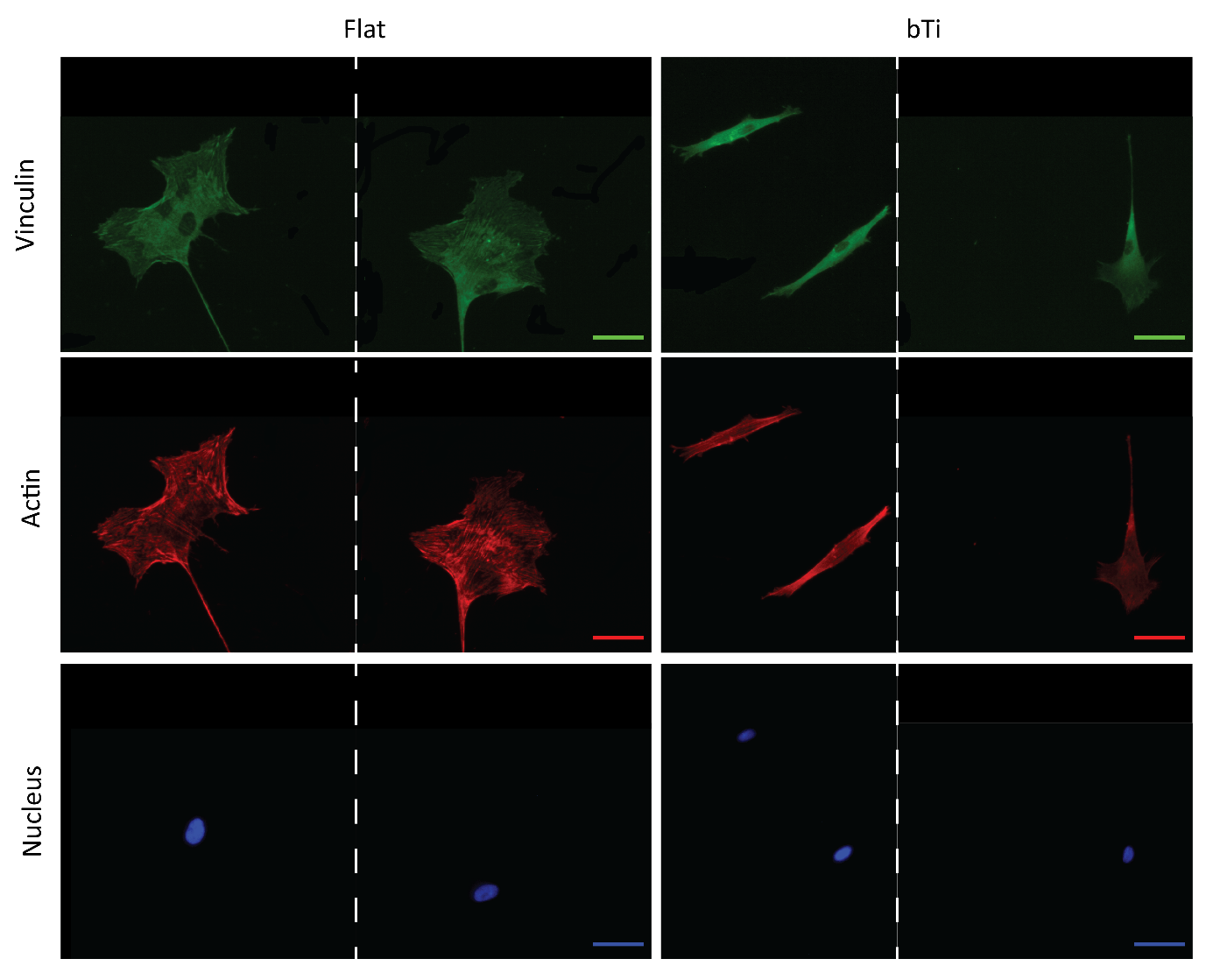


**Figure S5.** The separated channels of the fluorescence images of YAP-inhibited hMSCs cultured on the flat Ti and bTi surfaces for 1 day. Scale bar = 100 µm.

## Part II: Mineralization assay

We used a commercial osteogenesis kit assay to assess mineralization by hMSCs cultured on the investigated surfaces. hMSCs (PT-2501, passage number 7, Lonza Bioscience, The Netherlands) were grown in MSCGM^™^ Mesenchymal Stem Cell Growth Medium (#PT-3001, Lonza Bioscience, The Netherlands). Upon reaching confluence, the cells were trypsinized and seeded on flat Ti and bTi samples (*n* = 3 per group) with a density of 1 × 10^4^ cells per sample in a 48 well-plate. The samples were sterilized before cell seeding as described in the manuscript. The α-MEM culture medium used for this experiment was supplemented with 2% penicillin/streptomycin and 10% fetal calf serum (all from Thermo Fisher Scientific, The Netherlands). From day 2 of the culture onward, 100 nM dexamethasone and 10 mM β-glycerophosphate were added to the culture medium. The medium was refreshed every 2 days and the cells were kept in culture for 28 days.

On day 28 of culture, the cells were washed with 1X PBS and were fixated using a 4% (v/v) formaldehyde solution for 15 min. After removing the fixative, cells were rinsed 3 times with distilled water (each time for 5 min). An osteogenesis assay kit (#ECM815, Millipore, The Netherlands) was used to perform alizarin red staining and quantification. According to the instructions of the manufacturer, the samples were first stained with alizarin red solution at room temperature for 20 min and were then washed 4 times with distilled water (each time for 5 min). Subsequently, 200 µl of 10% acetic acid was added to each sample, which was then incubated for 30 min to loosen the cell monolayer. The monolayer was then scraped using cell scraper and moved to microcentrifuge tubes. The tubes were vortexed for 30 seconds and heated to 85 °C for 10 min. The tubes were then cooled on ice for 5 min and centrifuged at 20000 g for 15 min. Finally, 75 µl of 10% ammonium hydroxide was added to each tube to adjust the pH to a range of 4.1-4.5. A volume of 100 µl from each sample was transferred to a 96 well-plate (in duplicate) and its optical density was measured at the 405 nm wavelength. An alizarin red standard curve prepared in parallel was used to determine the alizarin red concentration in each sample.

The results indicated that the bTi surfaces significantly enhance mineralization compared to flat Ti surfaces (Figure S6).


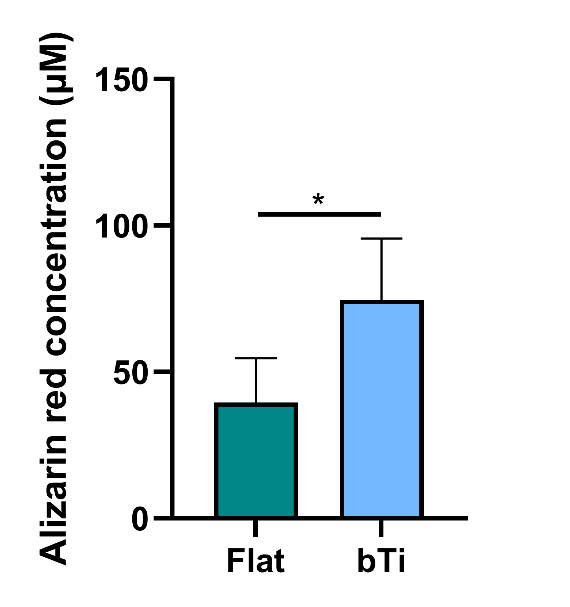


**Figure S6.** The evaluation of mineralization by the hMSCs cultured on flat Ti and bTi surfaces for 28 days. Unpaired *t*-test with Welch’s correction. * *p* < 0.05.

## Part III: Additional information on nuclear/cytoplasmic YAP


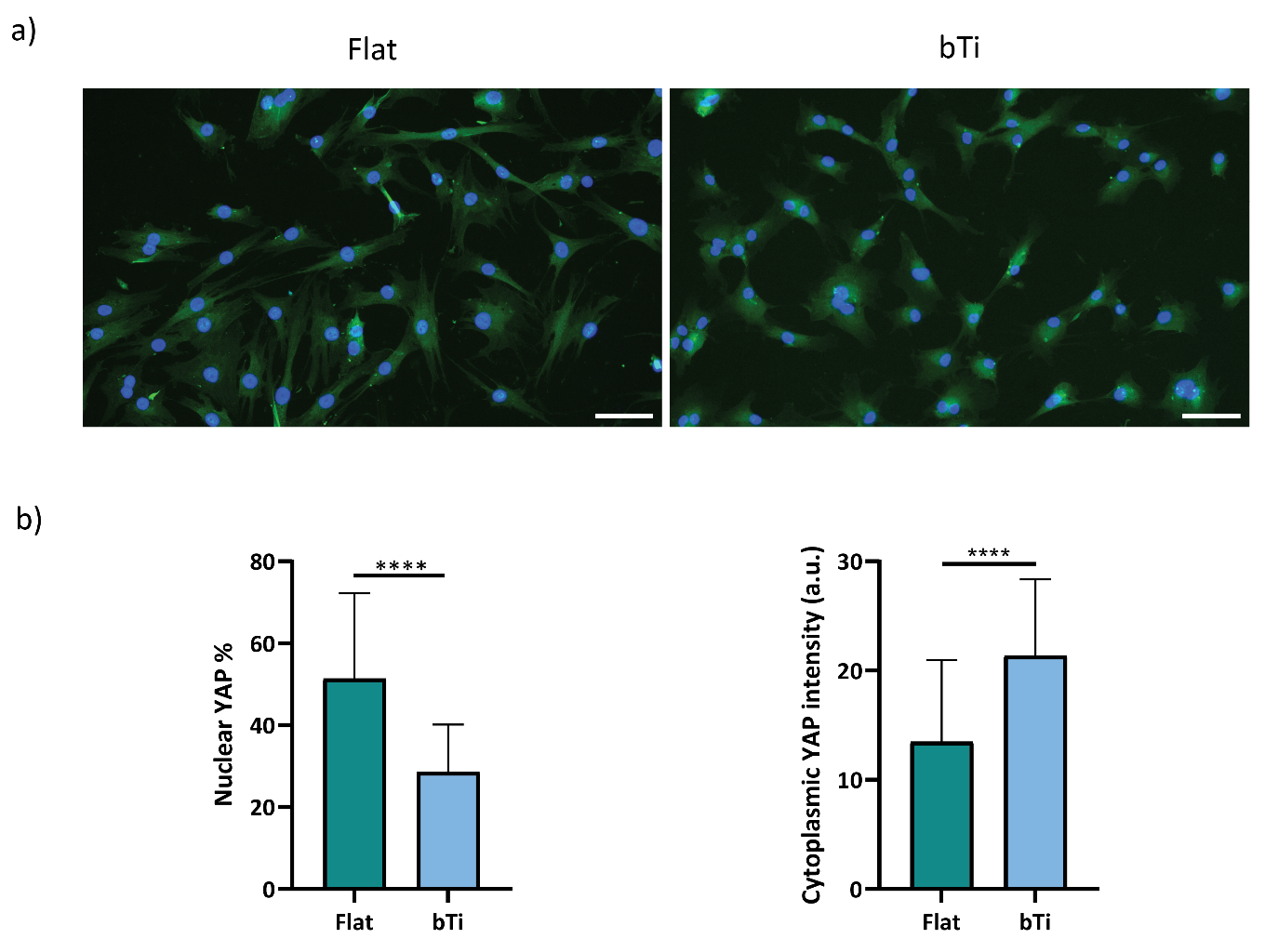


**Figure S7.** a) The representative merged images of the cells stained for nucleus and YAP on the flat Ti and bTi surfaces after 1 day of culture. Scale bar = 100 µm. b) Further information regarding the expression of YAP in the hMSCs. The nuclear YAP percentage in the cells residing on flat Ti surfaces was significantly higher than those seeded on the bTi surfaces. However, similar to the nuclear YAP, the signal intensity of cytoplasmic YAP was significantly higher on the bTi nanopillars. Mann-Whitney test. **** *p* < 0.0001.

**Part IV: qPCR**

We performed qPCR to investigate the expression of FAK, ROCK, and YAP genes in hMSCs cultured on flat Ti and bTi surfaces after 1 and 7 days of culture. Cells were grown as described in Part II and seeded on flat Ti and bTi samples (*n* = 3 per group) with a density of 1 × 10^5^ cells per sample in a 48 well-plate. At the desired time points, cells were washed with PBS and then 0.5 ml TRIzol RNA isolation reagent (Thermo Fisher Scientific, USA) was added to each well. After collecting the cells lysate in Eppendorf tubes, 100 µl chloroform was added to each tube and mixed by shaking. The tubes were kept on ice for 10 min and then spun down for 20 min at 4 °C and at 12900 RPM. Next, the transparent liquid phase of each tube was collected and mixed with 300 µl isopropanol. This mixture was spun down for 30 min at 4 °C and at 12900 RPM, and then three rounds of spinning down for 3 min at 4 °C was done: the first two rounds after adding 750 µl 70% ethanol and the last round after adding 100% ethanol. The tubes were then left to air dry and finally, the isolated RNA was suspended in 12 µl RNase-free water. After measuring the amount of isolated RNA, cDNA synthesis and qPCR were done according to previously established protocols at our collaborators’ lab at Erasmus MC University Medical Center. The primers used in this experiment are listed in Table S1 and GAPDH was used as the housekeeping gene.

**Table S1.** List of primers used for qPCR analysis

| Gene | Forward | Reverse |
| --- | --- | --- |
| PTK2 (FAK) | TTCAGTGCCTTCTGCAGTTTC | GCTAGGTATCTGTCATATTCTCCC |
| ROCK1 | GACATGCAAGCGCAATTGGT | CTTTGCTGGCCAACTGCATC |
| ROCK2 | TCACAAGGGGGTGATGGTTT | TGGAGTATCCCCCACTAGCA |
| YAP | TGGATGGGAACAAGCCATGA | CTCTGGTTCATGGCAAAACGA |
| GAPDH | CCGCATCTTCTTTTGCGTCG | CCCAATACGACCAAATCCGTTG |

The results indicated no significant difference in FAK and ROCK gene expression between flat Ti and bTi surfaces. However, a significantly higher mRNA expression of YAP was found on flat Ti surfaces on day 1 of culture (Figure S8). The significant dependency of cells on the activity of FAK and ROCK to express Runx2 when seeded on bTi nanopillars could be attributed to the events they undergo at the protein level. In this study, the inhibitor of FAK inhibited its phosphorylation on Tyr397 and the inhibitor of ROCK competed with ATP for binding to the catalytic sites of both ROCK1 and ROCK2. Therefore, we hypothesize that while interfacing with bTi nanopillars does not affect the gene expression, it alters the phosphorylation of FAK and the binding of ATP to ROCK in hMSCs. Further investigations of these events using advanced microscopy techniques within a biochemical perspective would be required to reveal the mechanism in full detail.


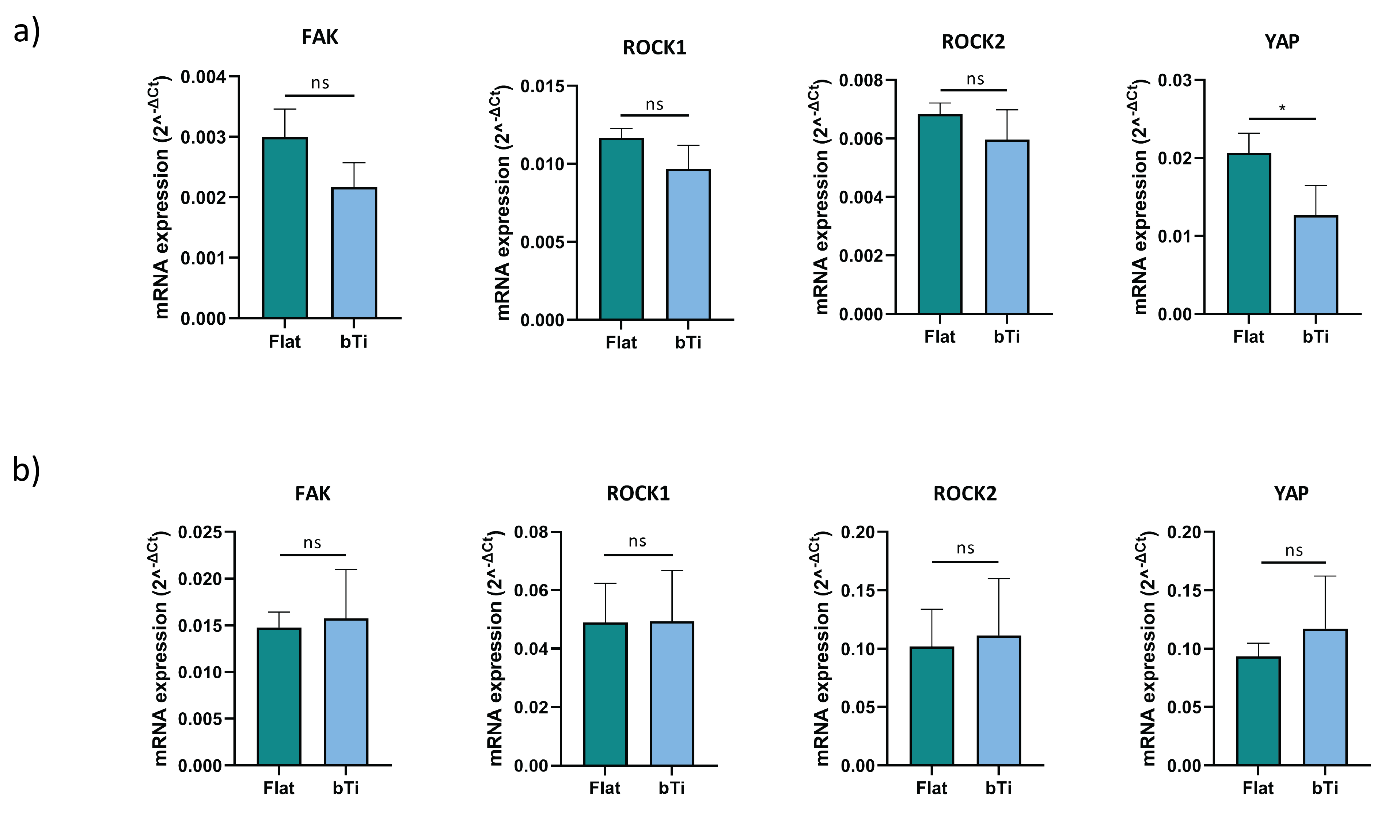


**Figure S8.** mRNA expression of FAK, ROCK1, ROCK2, and YAP in hMSCs cultured on flat Ti and bTi surfaces on a) day 1 and b) day 7 of culture.
